# Overexpression of *SPP1* is a prognostic indicator of immune infiltration in lung adenocarcinoma

**DOI:** 10.18632/aging.205526

**Published:** 2024-02-07

**Authors:** Binbin Li, Xue Li, Qingfeng Yang, Yiyang Jiang, Qianwen Zhang, Jingtao Zhang, Wenqiang Cui, Fei Xu

**Affiliations:** 1 College of Traditional Chinese Medicine, Shandong University of Traditional Chinese Medicine, Jinan 250014, China; 2 College of Acupuncture and Massage, Shandong University of Traditional Chinese Medicine, Jinan 250014, China; 3 Department of Pneumology, Affiliated Hospital of Shandong University of Traditional Chinese Medicine, Jinan 250014, China; 4 Department of Neurology, Affiliated Hospital of Shandong University of Traditional Chinese Medicine, Jinan 250014, China

**Keywords:** SPP1, LUAD, tumor-infiltrating immune cells, biomarker, prognosis

## Abstract

Objective: The extracellular phosphoprotein, secreted phosphoprotein 1 (SPP1), plays a crucial role in various tumors and regulating the immune system. This study aimed to evaluate its prognostic value and relationship to immune infiltration in lung adenocarcinoma (LUAD).

Methods: In the TCGA and GEO datasets, the information on clinic and transcriptome analysis of SPP1 in non-small-cell lung cancer (NSCLC) was examined accordingly. The association of SPP1 expression with overall survival and clinicopathologic characteristics was investigated by univariate and multivariate analysis. CancerSEA database was utilized to investigate the role of SPP1 at the cellular level by single-cell analysis. Additionally, the CIBERSORT algorithm was utilized to assess the correlation among the immune cells that infiltrated.

Results: NSCLC tissues exhibited a notable rise in SPP1 expression compared with that of normal tissues. Furthermore, the overexpression of SPP1 was substantially associated with clinicopathological features and unfavorable survival outcomes in individuals with LUAD, whereas no such correlation was observed in lung squamous cell carcinoma. Immune cells that infiltrate tumors and their corresponding genes were associated with SPP1 expression levels in LUAD.

Conclusions: SPP1 is a reliable indicator for assessing LUAD immune infiltration status and prognosis. With this approach, SPP1 can help earlier LUAD diagnosis and act as a possible immunotherapy target.

## INTRODUCTION

Over the past decade, lung cancer has emerged as the predominant form of cancer detected globally and the primary reason for mortality. According to current statistics, there are approximately 20,000 lung cancer cases per year, with mortality of 17,600 cases annually [1, 2]. Non-small-cell lung cancer (NSCLC) represents approximately 85% of all lung cancer cases [2]. In NSCLC, lung adenocarcinoma (LUAD) and lung squamous cell carcinoma (LUSC) are the most common subtypes [3]. However, over the last 20–30 years, LUAD has replaced LUSC as the most common histological type [4]. Additionally, lymph node metastasis is more common in LUAD than LUSC [5]. Therefore, more advanced research is required to develop effective treatment strategies, including early diagnosis and treatment with reliable targets.

Secreted phosphoprotein 1 (*SPP1*), which encodes osteopontin (OPN), was initially named due to its discovery in bone tissue. OPN is widely expressed in various tissues and cell types [6], including bone, kidney, and lung [7]. It plays crucial functional and regulatory roles under physiological and pathological conditions [8, 9]. The *SPP1* family exhibits specific binding capabilities and activates matrix metalloproteinases (MMPs) which are notable regulatory factors in tumors [10]. The secretion of this highly acidic phosphoprotein serves various purposes, such as promoting bone regeneration, facilitating angiogenesis, aiding in cell adhesion and migration, and contributing to inflammation [11]. In prostate [12], cervical [13, 14], breast [15], liver [16], and other cancers, *SPP1* is substantially expressed and correlated with clinical stage and prognosis [17]. However, the utilization of *SPP1* as a standalone prognostic indicator in LUAD remains unclear. Therefore, in this study, we aimed to investigate whether *SPP1* has prognostic significance in this disease.

Given the identified role of *SPP1* in other cancers and its demonstrated status as a biomarker for prognosis, this study sought to examine the expression of *SPP1* in LUAD, determine its relationship with clinical manifestations and prognosis, and provide a novel reference for the diagnosis and prognosis of LUAD combining bioinformatics and molecular biology.

## RESULTS

### Selection of DEGs associated with immune prognosis in NSCLC

**Figure 1:**
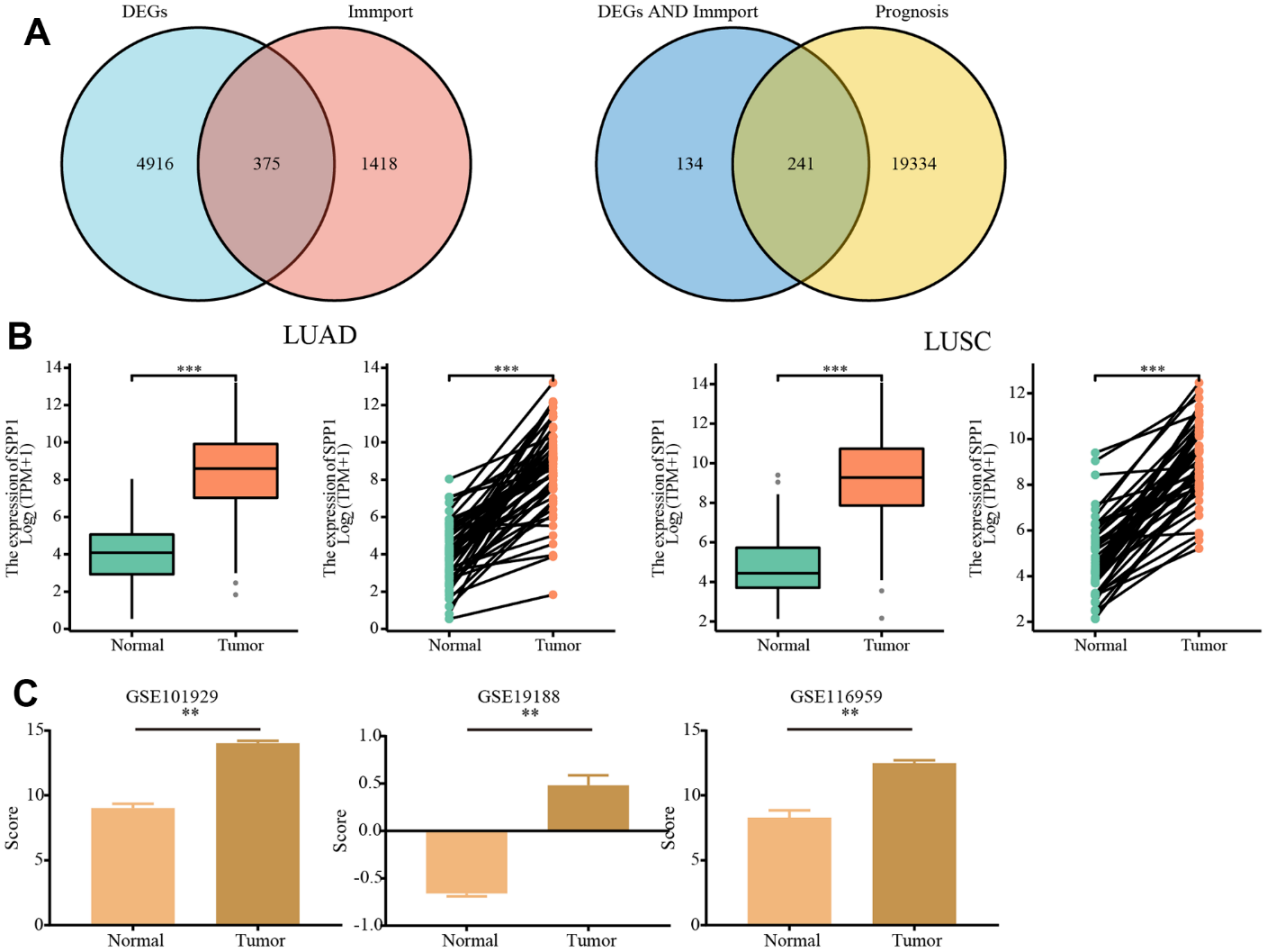
**Analysis of the expression of SPP1 based on TCGA and GEO databases.** (**A**) A Venn diagram of 241 immune- and prognosis-related differentially expressed genes. (**B**) According to the TCGA database differential expression of SPP1 in LUAD and LUSC versus normal tissues. (**C**) Differential expression of SPP1 based on GEO database GSE101929 (Normal=34, Tumor=32), GSE19188 (Normal=65, Tumor=91) and GSE116959 (Normal=11, Tumor=57). ^**^p<0.01, ^***^p<0.001.

In total, 5291 DEGs were identified, of which 241 were identified as being immune- or prognosis-related based on the ImmPort and TCGA database (Figure 1A). Out of the total, 47 genes were found to have a strong correlation with immune responses and prognosis, as determined by their |log2fc| (Supplementary Table 1), including *SPP1* (p=0.037). Due to its involvement in diverse biological processes, *SPP1* has been recognized as a significant contributor to numerous cancer types [18]. Consequently, *SPP1* has been chosen for further comprehensive examination.

### Unregulated expression of SPP1 in NSCLC

To clarify the expression pattern of *SPP1* in NSCLC, the transcriptomic data from TCGA was examined to analyze the expression of *SPP1* in normal, LUAD, and LUSC tissues. Figure 1B demonstrated a notable up-regulation of *SPP1* in LUAD and LUSC tissues when compared with normal tissues (all p < 0.001). A similar analysis was conducted on the GSE101929, GSE19188, and GSE116959 datasets. The analysis of *SPP1* expression in NSCLC, in comparison to normal tissues, also revealed a significant over-expression of *SPP1* (all p < 0.01; Figure 1C). Hence, it could be concluded that *SPP1* exhibited elevated levels of expression in NSCLC.

### Clinicopathological features of SPP1 in NSCLC

**Figure 2:**
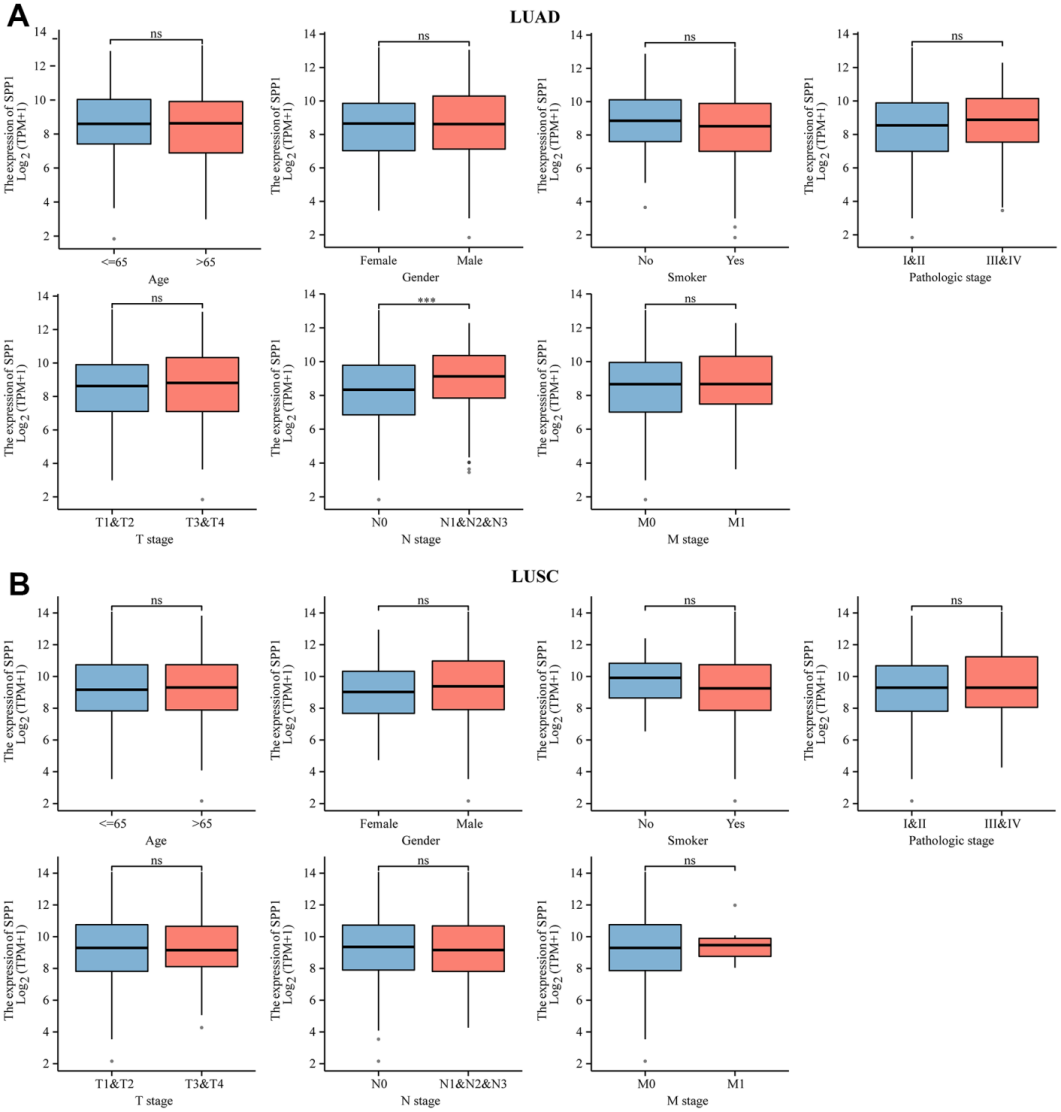
**Association between clinical-pathological characteristics and SPP1 mRNA levels in NSCLC.** Comparative analysis of SPP1 expression level of clinical-pathological characteristics in (**A**) LUAD and (**B**) LUSC. ns: no statistical significance, ^***^p<0.001.

The link between elevated *SPP1* expression and NSCLC led to the use of KW analysis to investigate *SPP1*-related clinicopathological features in LUAD and LUSC. In Figure 2A, a strong association was found between *SPP1* and the N stage of LUAD (p < 0.001), whereas no notable correlation was detected with other clinicopathological characteristics. Conversely, Figure 2B indicated no correlation between *SPP1* and the clinicopathological features of LUSC.

### Effect of SPP1 expression levels on prognosis in NSCLC

**Figure 3:**
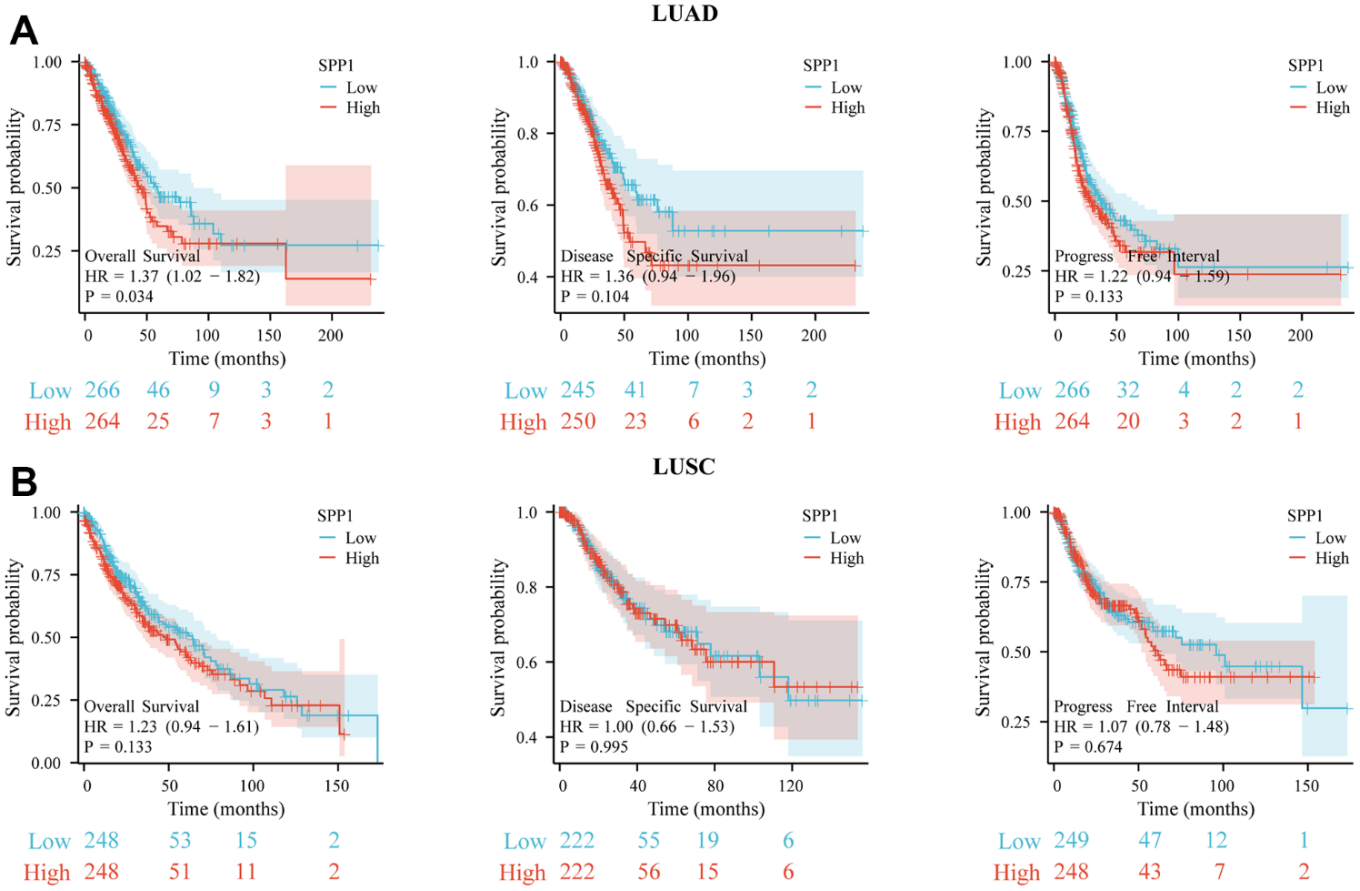
**Survival analysis for different expression of SPP1.** The prognostic impact of SPP1 on OS, DSS, and PFI in (**A**) LUAD and (**B**) LUSC. All gene sets were significantly enriched at nominal p-value <0.05.

To examine the influence of *SPP1* expression on the outlook of NSCLC, survival analysis curves were utilized to assess the overall survival (OS), disease-specific survival (DSS), and progression-free interval (PFI) of individuals diagnosed with LUAD and LUSC. Figure 3A illustrated that the OS of patients with LUAD was diminished in the presence of high expression of *SPP1* (HR 1.37 (1.02–1.82), p=0.034). However, the association between *SPP1* expression levels and DSS (HR 1.36 (0.94–1.96), p=0.104) and PFI (HR 1.22 (0.94–1.59), p=0.133) in patients with LUAD did not reach statistical significance. According to Figure 3B, there were no notable variances observed in the OS (HR 1.23 (0.94–1.61), p=0.133), DSS (HR 1.00 (0.66–1.53), p=0.995), or PFI (HR 1.07 (0.78–1.48), p=0.674) among LUSC individuals with elevated and reduced *SPP1* levels. Consequently, it could be concluded that elevated *SPP1* expression impacted the OS of patients with LUAD adversely, whereas the impact on the survival of patients with LUSC did not show statistical significance.

### Relationships between SPP1 expression and clinicopathological features of LUAD

As stated above, separate research on LUAD was initiated since the correlation of *SPP1* with LUSC was not significant. Considering the clinical importance of *SPP1* in LUAD, an examination of the detailed clinical attributes of patients with LUAD was initiated. Table 1 displayed a summary of clinicopathological features of 535 patients, encompassing age, gender, smoking habits, Tumor Node Metastasis (TNM) stage, pathological stage, tumor location, and primary effectiveness. Based on chi-square tests, *SPP1* showed a significant correlation with N stage (p=0.002) and primary efficacy assessment (p=0.025), while no significant correlation was found with other clinicopathological factors. This suggested that *SPP1* expression varied among the different LUAD N stages (N0, N1, N2 and N3). Patients with different efficacy evaluation outcomes also showed significantly different *SPP1* expression levels.

**Table 1:** Relationship between SPP1 expression and clinicopathological features of LUAD in TCGA.

**Characteristics**	**Low SPP1**	**High SPP1**	**p-value**
	**No. (%)**	**No. (%)**	
Age, n (%)			0.791
<=65	130 (25.2%)	125 (24.2%)	
>65	129 (25%)	132 (25.6%)	
Gender, n (%)			0.968
Female	142 (26.5%)	144 (26.9%)	
Male	125 (23.4%)	124 (23.2%)	
Smoker, n (%)			0.159
No	31 (6%)	44 (8.4%)	
Yes	227 (43.6%)	219 (42%)	
T stage, n (%)			0.220
T1	98 (18.4%)	77 (14.5%)	
T2	133 (25%)	156 (29.3%)	
T3	24 (4.5%)	25 (4.7%)	
T4	10 (1.9%)	9 (1.7%)	
N stage, n (%)			**0.002**
N0	192 (37%)	156 (30.1%)	
N1	36 (6.9%)	59 (11.4%)	
N2	30 (5.8%)	44 (8.5%)	
N3	0 (0%)	2 (0.4%)	
M stage, n (%)			1.000
M0	176 (45.6%)	185 (47.9%)	
M1	12 (3.1%)	13 (3.4%)	
Pathologic stage, n (%)			0.200
Stage I	158 (30%)	136 (25.8%)	
Stage II	57 (10.8%)	66 (12.5%)	
Stage III	35 (6.6%)	49 (9.3%)	
Stage IV	13 (2.5%)	13 (2.5%)	
Anatomic neoplasm subdivision, n (%)			0.350
Left	96 (18.5%)	109 (21%)	
Right	162 (31.2%)	153 (29.4%)	
Primary therapy outcome, n (%)			0.025
PD	28 (6.3%)	43 (9.6%)	
SD	24 (5.4%)	13 (2.9%)	
PR	5 (1.1%)	1 (0.2%)	
CR	173 (38.8%)	159 (35.7%)	

### Association between SPP1 expression and clinical prognosis in LUAD

The KM approach was utilized to examine the association between different clinicopathological characteristics and *SPP1* mRNA levels in LUAD, thereby evaluating its clinical prognostic significance. In elderly male patients aged 65 and above, as well as those with T stage T3, the research uncovered a noteworthy correlation between the expression of *SPP1* mRNA and the clinical prognosis (Table 2). This finding indicated that T staging, specifically T stage T3, was affected by clinical factors, such as *SPP1* mRNA expression, leading to variations in survival time among patients with LUAD and ultimately impacting clinical outcomes. Therefore, the objective was to investigate the prognostic impact of *SPP1* on LUAD in more detail.

**Table 2:** Correlation of SPP1 expression and clinical prognosis in LUAD with different clinicopathological factors by KM.

**Clinicopathological characteristics**		**No.**	**Hazard ratio**	**p-value**
Age				
	≤65	255	1.18(0.77-1.81)	0.437
	>65	261	1.54(1.03-2.30)	**0.036**
Gender				
	Female	286	1.62(1.08-2.42)	**0.02**
	Male	249	1.12(0.74-1.69)	0.601
Smoke				
	No	75	1.09 (0.49-2.40)	0.834
	Yes	446	1.28 (0.93-1.77)	0.127
T stage				
	T1	175	1.32 (0.72-2.42)	0.366
	T2	289	0.97 (0.67-1.42)	0.89
	T3	49	2.61 (1.01-6.71)	**0.047**
	T4	19	1.45 (0.46-4.61)	0.528
N stage				
	N0	348	1.03(0.68-1.56)	0.878
	N1	95	0.71 (0.41-1.22)	0.212
	N2	74	1.78(0.95-3.34)	0.073
	N3	2	---	---
M stage				
	M0	361	1.13 (0.81-1.59)	0.472
	M1	25	2.89 (0.90-9.32	0.076
Stage				
	I	294	1.00(0.63-1.61)	0.992
	II	123	0.86(0.50-1.48)	0.586
	III	84	1.78(0.99-3.22)	0.054
	IV	26	2.48(0.84-7.36)	0.102

### Prognostic analysis of LUAD clinical subgroups

**Figure 4:**
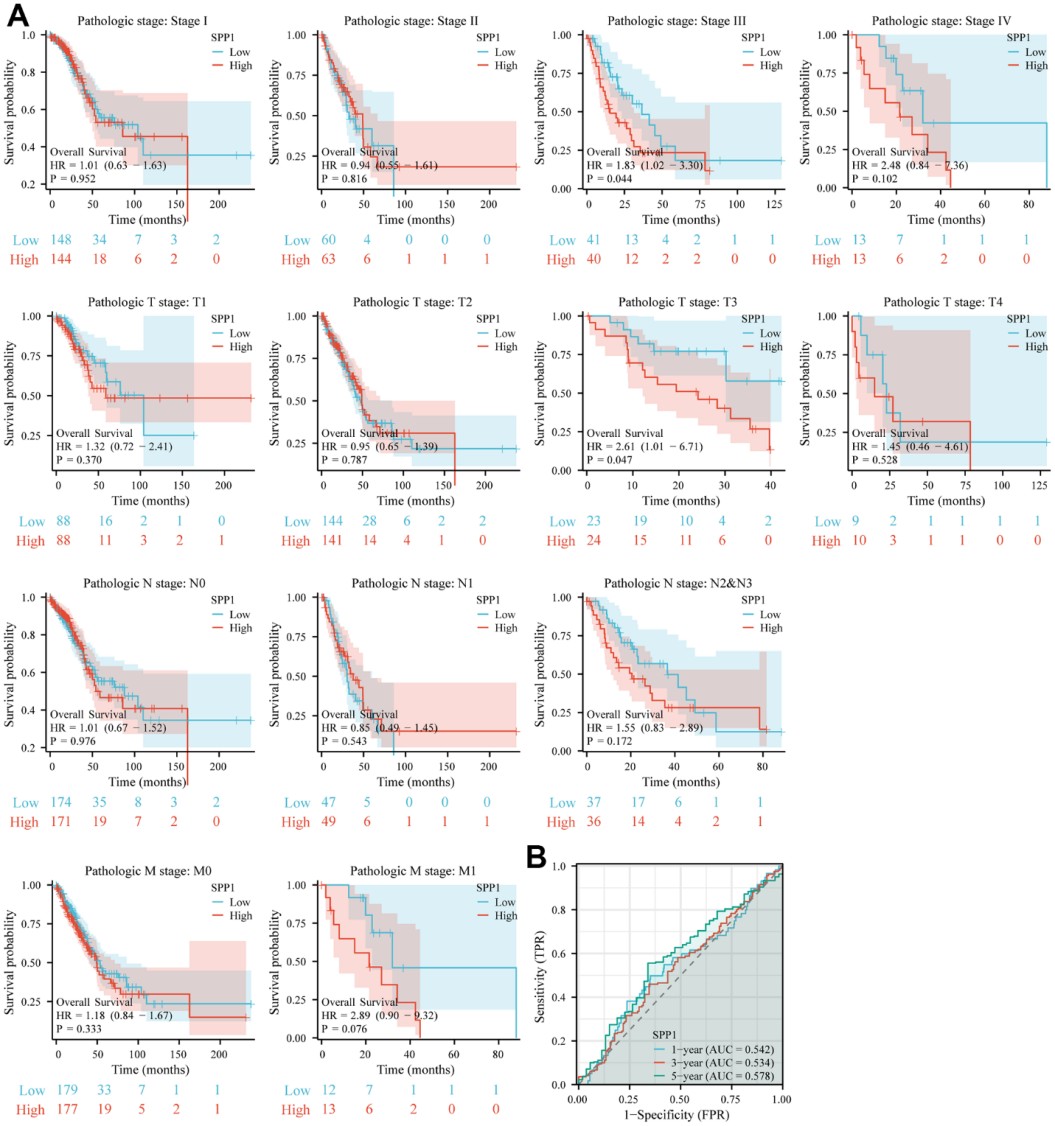
**The predictive significance of SPP1 in various subcategories.** (**A**) Significant association between elevated SPP1 expression and unfavorable overall survival was observed among different subgroups. (**B**) ROC curve of SPP1 expression at 1-, 3- and 5-year OS.

Further analysis was conducted to examine the correlation between the OS of LUAD and *SPP1* among different clinicopathological subgroups. Subgroup analysis showed that the higher expression of *SPP1* in pathological stage III (HR 1.83 (1.02–3.30), p=0.044) and T stage T3 (HR 2.61 (1.01–6.71), p=0.047) patients was statistically correlated with worse OS, as shown in Figure 4A. Using the ROC curves analysis, the predictive accuracy of *SPP1* for LUAD was evaluated in terms of 1-, 3-, and 5-year OS (Figure 4B). Area Under the Curve (AUC) > 0.5 suggested that *SPP1* served as a reliable prognostic indicator for LUAD patients, predicting outcomes at 1-, 3-, and 5-year intervals. The AUC under the ROC curves were 0.542, 0.534, and 0.578 in several. The findings demonstrated that *SPP1* exhibited slightly superior predictive capability for 1-, 3-, and 5-year survival compared to random prediction, thereby validating the accuracy of the diagnostic prediction. Therefore, the over-expression of *SPP1* was considered a risk factor for worse prognosis.

### Connection between SPP1 expression and OS among LUAD patients by univariate and multivariate methods

Further analysis was prompted by the significant correlation between *SPP1* and OS in LUAD patients, leading to the need for univariate and multivariate analysis. Through the analysis presented in Table 3, significant associations were observed between *SPP1* and various stages of LUAD. During the univariate analysis, *SPP1* showed connections with T3 and T4, N1, N2, and N3, M1, pathological III and IV stages, and high levels of *SPP1* expression. Univariate analysis revealed a significant association between the individual predictors and the survival times of patients with LUAD. After accounting for the interplay between multiple predictors and survival times, the multivariate analysis indicated that the T3 and T4 stages and the N1, N2, and N3 stages were correlated with patient OS. The findings of this study highlight the possibility of enhancing precision and inclusiveness in prognostic prediction. Notably, clinicopathological stage and a high *SPP1* expression level emerged as significant survival indicators and, following a comprehensive analysis of multiple factors, the latter portion of clinicopathological stage was identified as an independent prognostic factor for OS. The results provided a more accurate and thorough forecast, and comprehension of *SPP1* as a standalone predictive element.

**Table 3:** Univariate and multivariate analysis of the correlation of SPP1 expression with OS among LUAD patients.

**Parameter**	**Total (No.)**	**Univariate analysis**		**Multivariate analysis**	
		**Hazard ratio (95% CI)**	**p-value**	**Hazard ratio (95% CI)**	**p-value**
Age					
<=65	255				
>65	261	1.223 (0.916-1.635)	0.172	1.298 (0.911-1.849)	0.149
Gender					
Female	280				
Male	246	1.070 (0.803-1.426)	0.642	0.988 (0.693-1.408)	0.946
T stage					
T1&T2	457				
T3&T4	66	2.317 (1.591-3.375)	**<0.001**	1.922 (1.197-3.089)	**0.007**
N stage					
N0	343				
N1&N2&N3	167	2.601 (1.944-3.480)	**<0.001**	2.055 (1.353-3.120)	**<0.001**
M stage					
M0	352				
M1	25	2.136 (1.248-3.653)	**0.006**	1.300 (0.671-2.520)	0.437
Pathologic stage					
I&II	411				
III&IV	107	2.664 (1.960-3.621)	**<0.001**	1.380 (0.826-2.304)	0.218
Smoker					
No	72				
Yes	440	0.894 (0.592-1.348)	0.591	0.977 (0.584-1.636)	0.930
SPP1					
Low	264				
High	262	1.360 (1.019-1.814)	**0.037**	1.213 (0.851-1.730)	0.286

### Roles of SPP1 in LUAD

**Figure 5:**
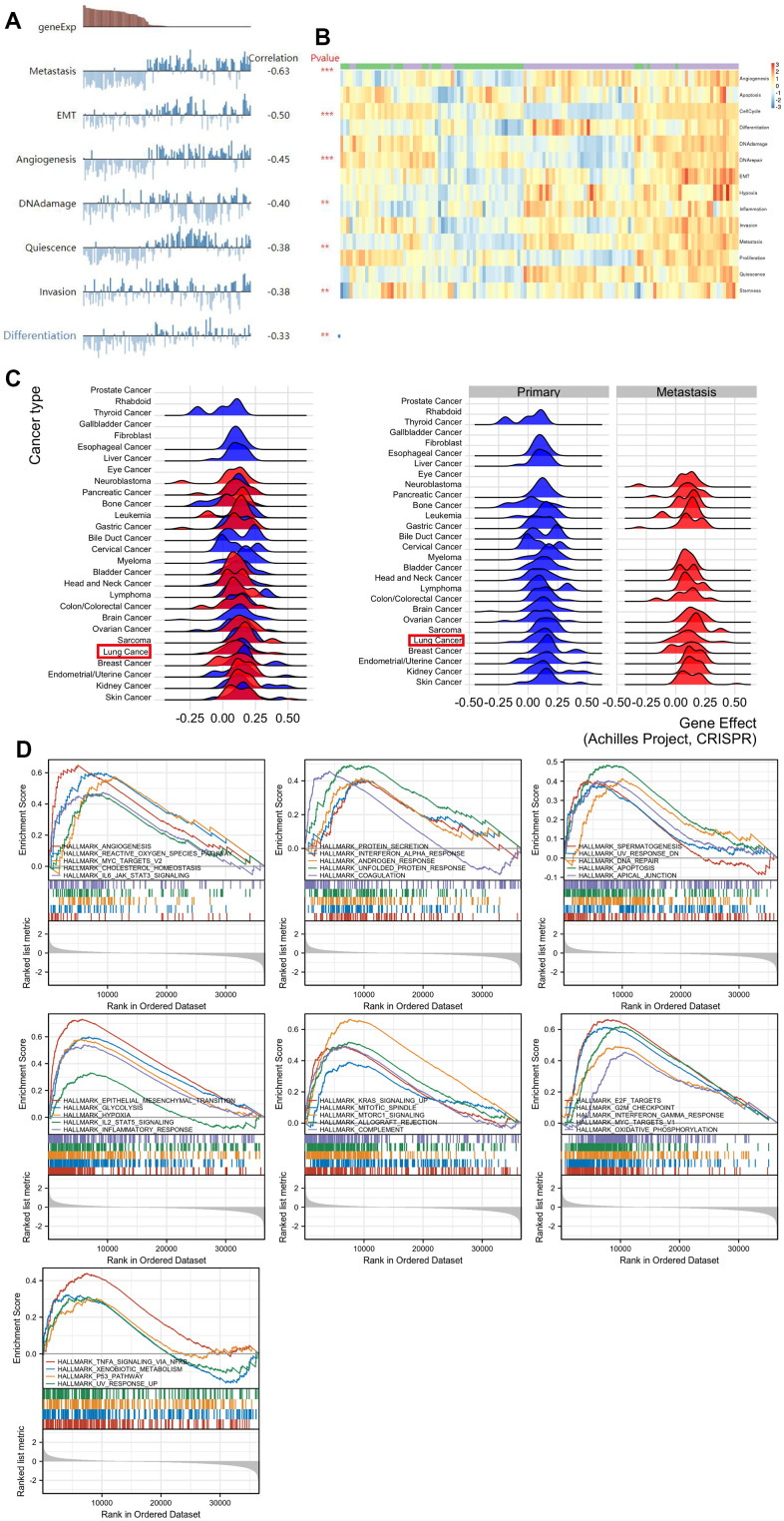
**Comprehensive analysis of the functional role of SPP1 in LUAD.** (**A**) Analysis of individual cells revealed multiple influence of SPP1, including cell metastasis, epithelial-to-mesenchymal transition, angiogenesis, DNA damage, cell dormancy, cancer cell invasion, and cellular differentiation. (**B**) Functional status profile showcasing the diverse activity of function states of LUAD cells. (**C**) Metastasis of lung cancer associated with SPP1 expression. (**D**) HALLMARK term analysis revealed in 34 positively correlated groups. ^**^p<0.01, ^***^p<0.001.

To enhance comprehension of *SPP1* manifestation and its fundamental mechanism in LUAD, single-cell analysis was conducted based on the CanerSEA database. The results indicated a negative correlation between *SPP1* and various cellular processes, including cell metastasis, epithelial-mesenchymal transition, angiogenesis, DNA damage, cell quiescence, cancer cell invasion, and cell differentiation (Figure 5A). Analysis of the entire heatmap enabled the identification of distinct activity patterns exhibited by different cells or cell groups in diverse functional states (Figure 5B). This comprehensive understanding aided in discerning disparities in functional states within cells, the level of activation of specific genes or pathways, and the heterogeneity among cell groups. A metastatic analysis based on the EMTome database revealed an association between *SPP1* and metastasis (Figure 5C). An analysis of GSEA on high *SPP1* groups was conducted to identify *SPP1*-associated signaling pathways. By the HALLMARK pathway enrichment analysis, the highly expressed *SPP1* phenotype was substantially enriched in 34 critical signaling pathways, one of which is EMT (Figure 5D). These results would help further explore the pathophysiological mechanisms of *SPP1*.

### Effect of SPP1 on epithelial-mesenchymal transition (EMT) in LUAD

**Figure 6:**
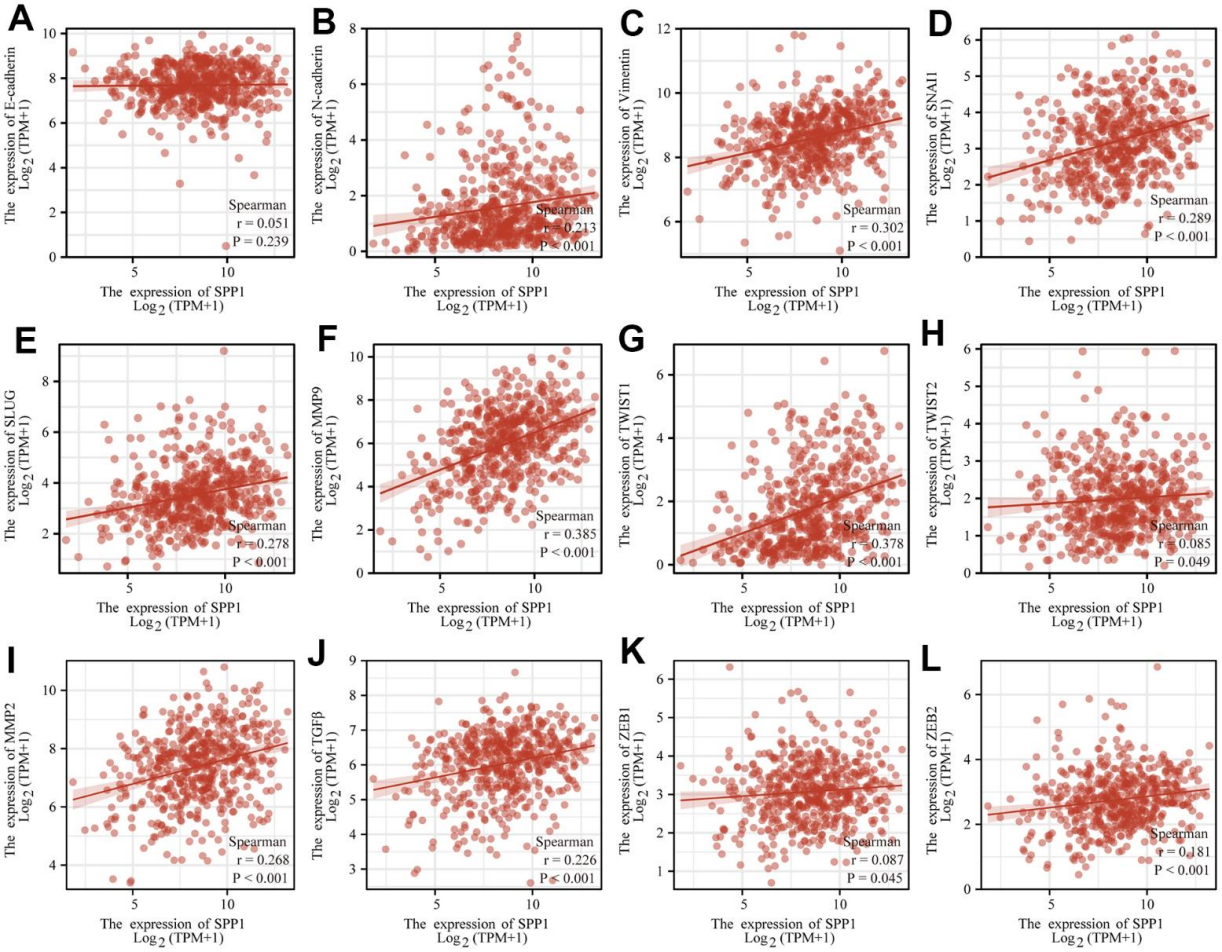
**SPP1 expression correlated with EMT signatures in LUAD.** SPP1 expression connected with (**A**) E-cadherin, (**B**) N-cadherin, (**C**) Vimentin, (**D**) SNAI1, (**E**) SLUG, (**F**) MMP9, (**G**) TWIST1, (**H**) TWIST2, (**I**) MMP2, (**J**) TGFβ, (**K**) ZEB1, and (**L**) ZEB2.

EMT is a crucial cellular process that was connected to *SPP1* in the above-mentioned study [19, 20]. To gain further insights into the mechanistic actions of *SPP1*, an analysis was conducted on the effects of *SPP1* as a marker on EMT (Figure 6). The activation of EMT, where epithelial cells experienced a transition and acquired mesenchymal properties, hence enhancing their motility and migratory potential, emerged as a crucial mechanism in the spread of cancer cells. Notably, Figure 6 demonstrated a correlation between the upregulation of *SPP1* expression and the heightened expression of mesenchymal cell markers (N-cadherin, vimentin). At the same time, a number of transcription factor families, including SNAI1, SLUG, TWIST1, TWIST2, ZEB1, and ZEB2, controlled how the EMT process was modulated. Additionally, proteolytic digestion was made easier by the overexpression of matrix metalloproteinases (MMP2 and MMP9). The expression of MMP was regulated by the EMT-related signal transduction pathway (TGF-β). Collectively, these results implied that *SPP1* may be involved in EMT pathways that were critical for the development of LUAD.

### Detection of SPP1 infiltrating immune cells correlates with its expression

**Figure 7:**
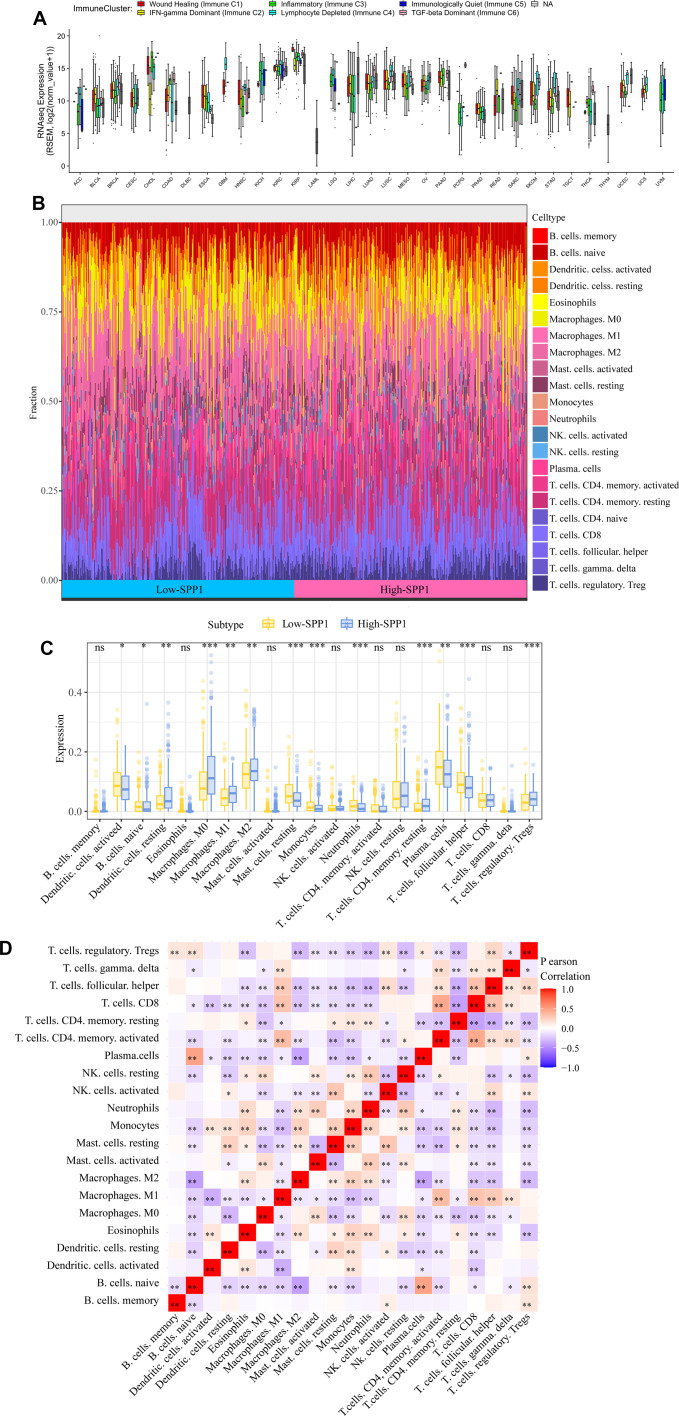
**Impact of SPP1 on immune cell infiltration and distribution in LUAD.** (**A**) Distinct immune clusters observed in different cancer types. (**B**) Immune landscape data of LUAD from different expression groups of SPP1. (**C**) Variations in the ratios of 21 different immune cell types between tumor samples with high and low SPP1 expression. (**D**) A heat map illustrated the spread of these immune-infiltrating cells within the tumor specimens. ns: no statistical significance, *p<0.05, **p<0.01, ***p<0.001.

It is widely recognized that the immune system is crucial in the fight against cancer [21]. The role of *SPP1* was investigated in the tumor microenvironment (TME) of LUAD by the EMTome database and CIBERSORT algorithm to analyze TCGA data and determine the landscape of immune cell infiltration in LUAD (Figure 7A, 7B). Subsequently, the specimens were categorized into two cohorts according to *SPP1* manifestation, and the analysis of immune cells distribution between these cohorts was conducted. In Figure 7C, which represented the high *SPP1* groups, a significant increase in the density of various immune cells was observed included M0, M1, and M2 macrophages, as well as resting memory CD4+ T cells and regulatory T cells (Tregs). The heat map analysis revealed a connection between 21 immune-infiltrating cells and tumor samples in the TCGA cohort, as indicated by the correlation observed (Figure 7D).

It was found that *SPP1* expression was correlated with 4 tumor-infiltrating immune cells by utilizing the TIMER database (Supplementary Figure 1A). We also identified relationships between *SPP1* and the expression of 28 TILs in human cancers, with significant correlations with 23 of these immune cells. (Supplementary Figure 1B).

This discovery showed that *SPP1* could play a special function in the immune cells’ invasion of LUAD.

### Immune-inhibitory and immunostimulatory factors associated with SPP1 expression

Co-expression analysis was carried out utilizing TISIDB to learn more about the relationship between *SPP1* and immunity. The use of immunomodulators in immunotherapies to target tumor cells and the TME around them has proven beneficial [22]. The goal was to determine how *SPP1* expression and the expression of immunomodulators—which included both immunosuppressants and immunostimulants—related to one another.

**Figure 8:**
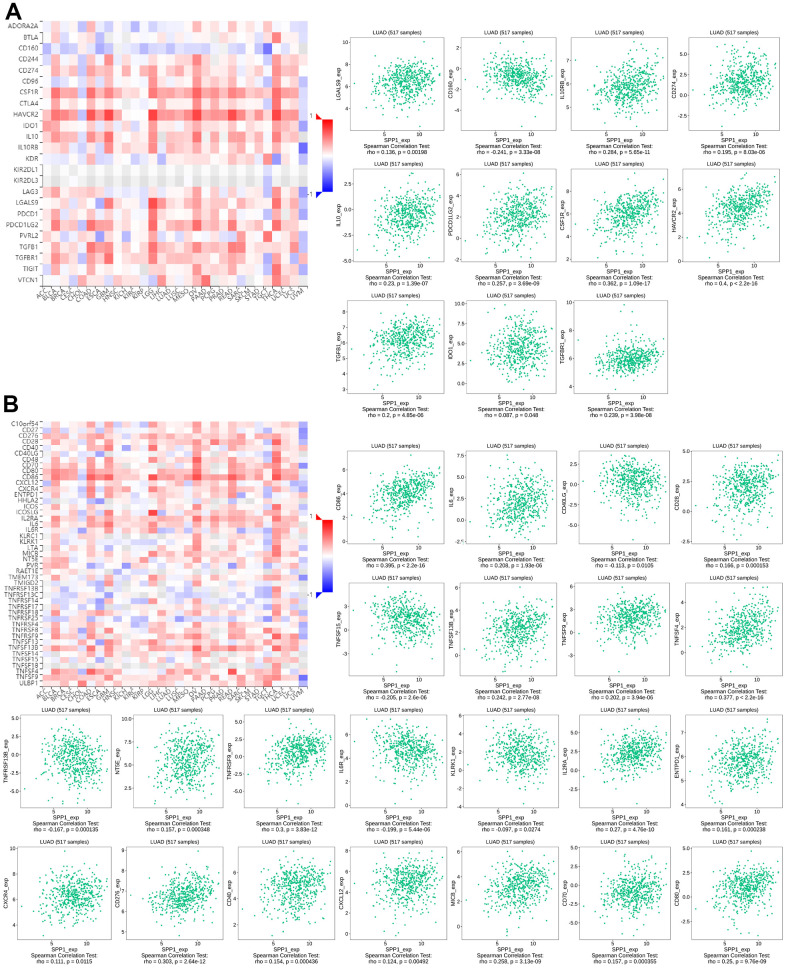
**Correlation between the levels of immune infiltration and SPP1 expression.** (**A**) Relationship between 23 immunosuppressants and SPP1 expression in LUAD. (**B**) Correlation between 46 immune enhancers and SPP1 expression in LUAD.

Figure 8A visually depicted the association between *SPP1* and the expression levels of 23 immunosuppressants in various human cancers, as obtained from the TISIDB database. There was a significant correlation between the expression of *SPP1* and 11 immunosuppressants, including CD274 (rho=0.195, p=8.03e–06), CSF1R (rho=0.362, p=1.09e–17), HAVCR2 (rho=0.4, p<2.2e–16) and TGFBR1 (rho=0.239, p=3.98e–08). The expression of *SPP1* could be regulated by immunosuppressive agents. Some immunosuppressants can inhibit *SPP1* production or inhibit *SPP1* expression in immune cells [23, 24].

The relationship between *SPP1* and the expression of 46 immune enhancers in human cancers from the TISIDB database was depicted in Figure 8B. A strong association was observed between *SPP1* expression and 22 of these enhancers, including TNFSF15 (rho=–0.205, p=2.6e–06) and IL6R (rho=–0.199, p=5.44e–06). Therefore, it was inferred that *SPP1* could serve as a target of immunostimulants or mediate the effects of immunostimulants.

The findings suggested that *SPP1* may have a function in modifying immunological responses by being engaged in the regulation of these immune modulators.

### Correlation analysis on LUAD to examine the relationship between SPP1 and associated genes and markers of immune cells

To better understand the connection between *SPP1* expression and immune cell infiltration in LUAD, Table 4 displayed the results of the correlation between *SPP1* expression and a number of markers related to immune infiltration. *SPP1* expression was correlated with the majority of immunological marker sets of monocytes, tumor-associated macrophage (TAM), M1 macrophage, M2 macrophage, Dendritic cell and Tregs in LUAD. Additionally, *SPP1* copy number alterations were examined in tumors with varying invasion levels. Notably, the arm-level deletion copy number variant of *SPP1* exhibited a significant association with CD4^+^ T cells, macrophages, and dendritic cells infiltration in LUAD (Supplementary Figure 2). Consequently, *SPP1* may modulate immune cell function through its ability to regulate marker gene expression.

**Table 4:** Correlation analysis between SPP1 and relate genes and markers of immune cells in LUAD by TIMER.

	**Gene markers**	**None**	**p-value**	**Purity**	**p-value**
		**Correlation**		**Correlation**	
CD8^+^ T cell	CD8A	0	0.9920	-0.059	0.1940
	CD8B	-0.012	0.7830	-0.055	0.2190
T cell (general)	CD3D	0.044	0.3140	-0.017	0.7080
	CD3E	-0.021	0.6410	-0.1	*
	CD2	0.032	0.4670	-0.035	0.4350
B cell	CD19	-0.045	0.3090	-0.113	*
	CD79A	0.033	0.4530	-0.021	0.6490
Monocyte	CD86	0.405	***	0.411	***
	CD115 (CSF1R)	0.376	***	0.37	***
TAM	CCL2	0.346	***	0.334	***
	CD68	0.319	***	0.313	***
	IL10	0.25	***	0.237	***
M1 macrophage	INOS (NOS2)	0.005	0.9140	-0.033	0.4630
	IRF5	0.194	***	0.176	***
	COX2 (PTGS2)	0.117	*	0.11	*
M2 macrophage	CD163	0.316	***	0.308	***
	VSIG4	0.379	***	0.374	***
	MS4A4A	0.331	***	0.332	***
Neutrophils	CD66b (CEACAM8)	-0.016	0.7090	-0.032	0.4720
	CD11B (ITGAM)	0.358	***	0.352	***
	CCR7	-0.056	0.2020	-0.134	*
Natural killer cell	KIR2DL1	-0.063	0.1540	-0.095	*
	KIR2DL3	-0.031	0.4780	-0.06	0.1810
	KIR2DL4	0.095	*	0.068	0.1300
	KIR3DL1	-0.07	0.1130	-0.107	0.0174
	KIR3DL2	0.005	0.9140	-0.018	0.6880
	KIR3DL3	0.044	0.3240	0.022	0.6310
	KIR2DS4	-0.049	0.2710	-0.081	0.0710
Dendritic cell	HLA-DPB1	0.094	*	0.055	0.2200
	HLA-DQB1	0.099	*	0.067	0.1370
	HLA-DRA	0.192	***	0.169	**
	HLA-DPA1	0.152	**	0.117	*
	BDCA-1 (CD1C)	0.052	0.2360	0.03	0.5120
	BDCA-4 (NRP1)	0.16	**	0.139	*
	CD11c (ITGAX)	0.209	***	0.237	***
Th1	T-bet (TBX21)	-0.075	0.0911	-0.142	*
	STAT4	0.09	*	0.04	0.3750
	STAT1	0.158	**	0.127	*
	IFN-g (IFNG)	0.062	0.1620	0.021	0.6350
	TNF-a (TNF)	0.173	***	0.151	**
Th2	GATA3	0.051	0.2450	-0.006	0.8960
	STAT6	-0.173	***	-0.168	**
	STAT5A	0.076	0.0864	0.036	0.4220
	IL-13	-0.044	0.317	-0.074	0.0989
Tfh	BCL6	0.016	0.7210	0.001	0.9850
	IL21	0.042	0.3440	0.01	0.8270
Th17	STAT3	0.011	0.8030	0.004	0.9220
	IL17A	-0.021	0.6390	-0.047	0.2940
Treg	FOXP3	0.146	**	0.097	*
	CCR8	0.164	**	0.13	*
	STAT5B	-0.062	0.1630	-0.074	0.0998
	TGF-β(TGFB1)	0.208	***	0.179	***
T cell exhaustion	PD-1(PDCD1)	0.067	0.1290	0.016	0.7240
	CTLA4	0.074	0.0937	0.019	0.6800
	TIM-3(HAVCR2)	0.406	***	0.407	***
	GZMB	0.127	*	0.09	*
	LAG3	0.052	0.2430	0.006	0.8960
	PDL1(CD274)	0.216	***	0.186	***

^*^p<0.05, ^**^p<0.01, ^***^p<0.001.

### Correlation between SPP1 expression and drug sensitivity

**Figure 9:**
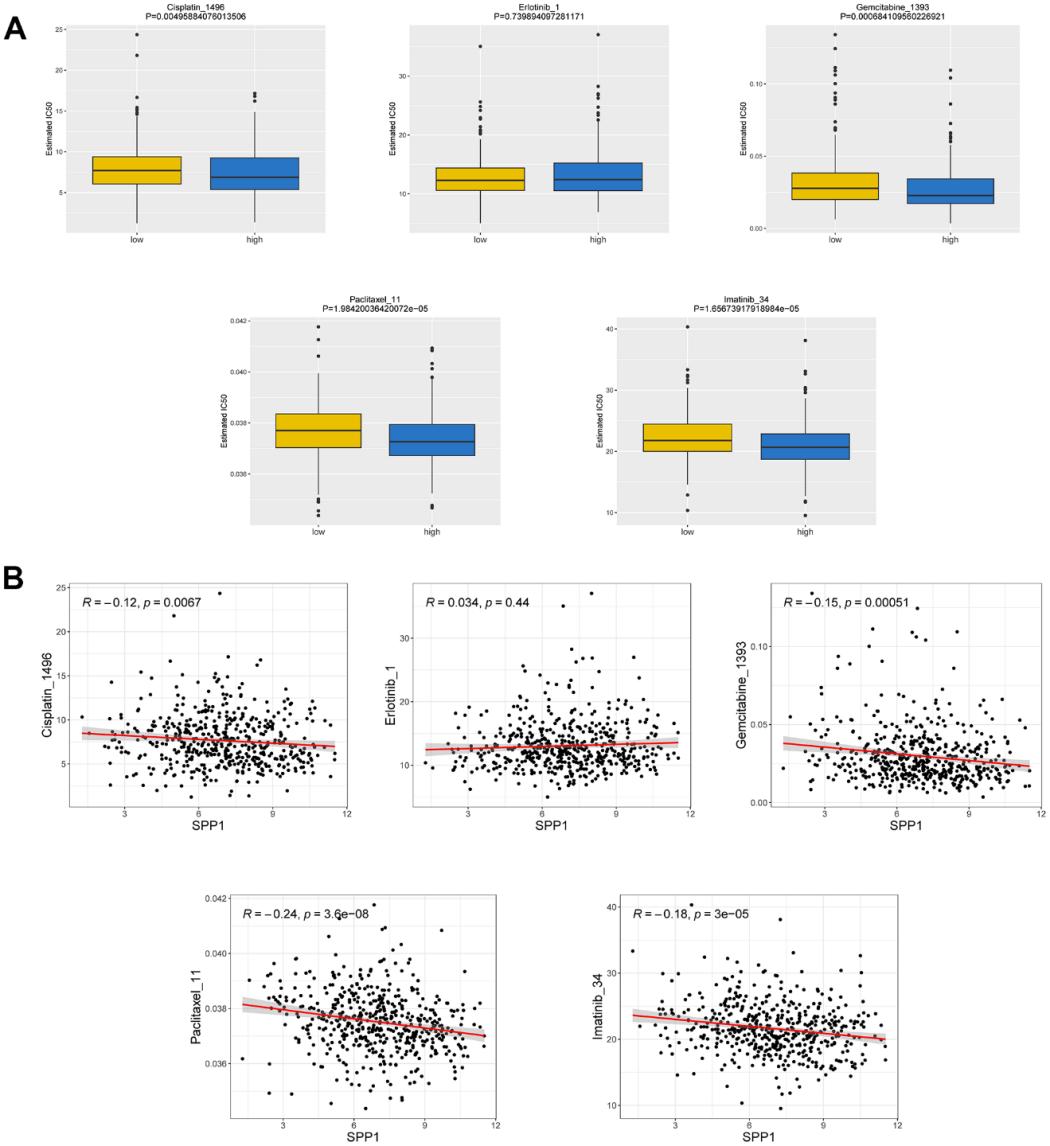
**Correlation between drug susceptibility and SPP1 expression.** (**A**) IC_50_ difference between groups with high and low expression of the SPP1. (**B**) Relationship between IC_50_ and SPP1.

Drug resistance has long been recognized as a major obstacle in LAUD management. To enhance the clinical efficacy of different treatments, it is imperative to subject different chemotherapy drugs to sensitivity analysis. In this study, we analyzed the sensitivity of 367 drugs in the GDSC database. Specifically, 510 samples in the TCGA-LUAD dataset were categorized into the high and low expression groups based on the expression levels of the *SPP1* gene, and subsequently, IC_50_ values were calculated. Remarkably, we observed that the IC_50_ values of 127 drugs exhibited significant disparities between the high and low *SPP1* expression groups, including notable drugs, such as gemcitabine and cisplatin (Figure 9). Spearman correlation analysis also showed a significant negative correlation between *SPP1* expression level and the two drugs, gemcitabine and cisplatin. This finding suggested that tumors with elevated *SPP1* expression levels show heightened sensitivity to gemcitabine and cisplatin, rendering them more likely to respond favorably to these treatments compared with counterparts with low *SPP1* expression levels. Moreover, these findings provided valuable insights for tailoring personalized treatments for individual patients with LUAD. However, further research is required to validate these observations and elucidate the underlying associated mechanisms.

## DISCUSSION

LUAD is a common cancer type distinguished by a substantial frequency and elevated fatality rate [25, 26]. In the treatment of LUAD, radiotherapy and chemotherapy remain the primary therapeutic modalities for patients with advanced and end-stage disease, whereas radical surgery is preferred for patients in early stages [27]. Therefore, it is crucial to investigate effective molecular targets to improve treatment results [28]. Such advancements will bring us closer to overcoming the formidable challenges associated with cancer. Nevertheless, the use of pharmaceutical interventions is considerably constrained by the presence of adenocarcinoma mutations and drug resistance, which impedes their widespread application [29, 30]. Therefore, it is crucial to identify new predictive biomarkers that can explore the underlying mechanisms of LUAD and support the advancement of therapeutic treatments.

*SPP1* gene refers to the gene encoding Osteopontin-1. OPN, which is secreted by tumor cells, osteoclasts, immune cells, and other cells, being a constituent of the extracellular matrix [31]. *SPP1* glycoprotein is involved in a variety of important physiological and pathological processes outside the cell, including cell migration, recruitment of inflammatory cells and tumor metastasis [32]. *SPP1* is extensively found in various human tissues and organs, highly expressed in numerous tumors, and linked to unfavorable prognosis [33]. It is secreted by diverse cancer cell types, playing a role in the initiation, advancement, spread, infiltration, and resistance to radiotherapy of tumors [27, 34]. Nevertheless, the precise workings of *SPP1* in LUAD are still not fully understood.

The major goal of this study was to investigate the role of *SPP1* in LUAD prognosis and immune response. *SPP1* was identified as one of the DEGs showing a strong correlation with immune responses and prognosis in NSCLC. Analysis of data from the TCGA databases revealed that NSCLC samples exhibited elevated levels of *SPP1* expression compared with normal tissues. These results were further validated using the GEO database. We also observed that elevated *SPP1* expression levels were significantly associated with clinicopathological features and unfavorable survival outcomes in patients with LUAD, whereas no such correlation was observed in patients with LUSC. Therefore, considering that the correlation between *SPP1* and LUSC was not significant, our subsequent analyses involved only LUAD. Furthermore, the detailed analysis of the relationship between *SPP1* expression and the clinical attributes of patients with LUAD showed a significant correlation between *SPP1* expression level and N stage and primary efficacy assessment, whereas no significant correlation was observed for other clinicopathological factors. For older male patients (aged ≥65 years) and those with disease at the T3 stage, our analysis showed a significant correlation between *SPP1* mRNA expression level and the clinical prognosis. Therefore, we further investigated the prognostic impact of SPP1 on LUAD. The prognostic analysis of clinical subgroups in LUAD showed that the higher *SPP1* expression level in patients with pathological stage III and T stage T3 of the disease was significantly associated with a worse OS. The ROC curves obtained in this regard showed that *SPP1* exhibited slightly superior predictive capability for 1-, 3-, and 5-year survival compared with random prediction, thereby validating the accuracy of the *SPP1* expression-based diagnostic prediction in LAUD. Moreover, both univariate and multivariate analyses provided additional evidence supporting *SPP1* expression as a significant predictor of survival, consistent with findings from previous studies [35]. Numerous research has shown a robust association between the manifestation of *SPP1* and tumor cells evolutionary progression, along with the microenvironment reprogramming [36–39]. Furthermore, it has been noted that the existence of molecular diversity within tumors is vital in the emergence of resistance to treatment and substantially affects the prognosis of patients. Notably, both experimental and human invasive lung cancers have exhibited an overexpression of *SPP1*, which has been associated with unfavorable survival outcomes [40, 41]. As a result, the increased expression of *SPP1* is commonly considered as a biomarker, suggesting an unfavorable prognosis in individuals diagnosed with LUAD.

Since *SPP1* showed association with OS in patients with LUAD, we performed further analyses, which revealed significant associations between *SPP1* expression level and various stages of LUAD. To elucidate the molecular mechanisms of *SPP1* in LUAD, CancerSEA and GSEA analyses were performed. Furthermore, we comprehensively analyzed the functional role of *SPP1* in LUAD. The results obtained showed a negative correlation between *SPP1* expression levels and various cellular processes, including metastasis, EMT, angiogenesis, DNA damage, cancer cell invasion, and cell differentiation. Therefore, our findings enhance comprehension regarding the role of *SPP1* and its fundamental mechanism in LUAD cells. Based on the EMTome database, a metastatic analysis revealed an association between *SPP1* and metastasis in lung cancer. According to gene enrichment analysis, *SPP1* is associated with 34 pathways, one of which is EMT. These results would help further explore the pathophysiological mechanisms of *SPP1*. Additionally, the upregulation of *SPP1* was involved in EMT pathways that were critical for the development of LUAD, including the heightened expression of mesenchymal cell markers (N-cadherin, vimentin), a number of transcription factor families (SNAI1, SLUG, TWIST1, TWIST2, ZEB1, and ZEB2) which controlled how the EMT process was modulated, the overexpression of matrix metalloproteinases (MMP2 and MMP9) and the EMT-related signal transduction pathway (TGF-β). Subsequently, the correlation was examined between the expression of *SPP1* and the infiltration of immune cells in LUAD.

This study found that a close association was observed between *SPP1* and immunomodulatory factors. Research has demonstrated that the host immune system can be regulated by *SPP1* in mouse macrophages and natural killer cells [42, 43]. A variation in the plasma level of OPN expressed by *SPP1* could impose an influence on cancer metastasis, which had a chemotactic effect on numerous immune cells and affected cell-mediated immunity [44]. In LUAD, the involvement of *SPP1* included the increase of PD-L1 levels, which subsequently affected the polarization of macrophages and aided in evading the immune system [45, 46]. Together, these findings suggested a potential involvement of *SPP1* in immunity. The analysis showed that immune cells that infiltrate tumors and TILs were associated with *SPP1* expression levels in LUAD. High levels of *SPP1* upregulated the immunosuppressive expression of LGALS9, IL10RB, CD274, IL10, PDCD1LG2, CSF1R, HAVCR2, TGFB1, IDO1, and TGFBR1. Simultaneously, *SPP1* downregulated CD40LG, TNFSF15, TNFRSF13B, IL6R, KLRK1, and other immune stimulants, indicating that *SPP1* played a role in the evasion of tumor immunity by controlling the immunosuppressive surroundings. These findings highlighted the possible function of *SPP1* in the TME of LUAD.

Furthermore, the expression of *SPP1* exhibited correlations with various factors, including tumor purity, macrophages, neutrophils, and DCs. Notably, *SPP1* expression was related to a number of markers related to immune infiltration and the arm-level deletion copy number variant of *SPP1* exhibited a significant association with CD4+ T cells, macrophages, and dendritic cells infiltration in LUAD. Previous research has also demonstrated the significance of *SPP1* as a chemokine in recruiting macrophages to glioblastomas, facilitating communication between tumor cells and the innate immune system, and potentially serving as a therapeutic target [47]. Furthermore, the tumor immune microenvironment was affected by *SPP1* as it increases the PD-L3 expression via the PI1K/AKT, JAK, and TGF-β signaling pathways [48]. Other studies have also provided a comprehensive analysis of the interplay between *SPP1* and its receptor, CD47, elucidating their inhibitory effects on angiogenesis through the antagonism of nitric oxide signaling in endothelial and vascular smooth muscle cells [49]. These studies supported the findings of this study related to the role of *SPP1* in the immune system of patients with LUAD.

In summary, we have demonstrated that *SPP1* is a valuable prognostic marker for LUAD which may prove beneficial for improved disease prediction and immunotherapy. However, the specific mechanism of action awaits verification, and further experimental studies, as well as clinical trials will be necessary.

This study offered a thorough examination of the predictive and immune relationship between *SPP1* and LUAD. Consequently, these findings have significant implications for the TME. Specifically, this investigation systematically investigates the impact of *SPP1* on tumor progression, prognosis, and immune in individuals diagnosed with LUAD. The findings of this research indicate that the increase in *SPP1* is involved in immune transmission and is strongly linked to an adverse prognosis in patients with LUAD. Thus, *SPP1* emerges as a promising biomarker for prognosticating human LUAD and represents a novel therapeutic target.

## MATERIALS AND METHODS

### Screening of differentially expressed genes (DEGs)

A total of 5291 DEGs were screened using transcriptome data from NSCLC (LUAD and LUSC) cells or tissues and normal tissue samples extracted from The Cancer Genome Atlas (TCGA) (https://portal.gdc.cancer.gov/). The statistical thresholds for DEG analysis were as follows: |log (fold change) | > 2 and adjusted p-value < 0.01. Additionally, duplicate gene names were eliminated. Therefore, 2483 immune-related genes were identified using the Immunology Database and Analysis Portal (ImmPort) database. Eventually, 1793 genes were retained for the study after excluding genes with identical symbol names. Subsequently, we investigated the overlap between DEGs and immune-associated genes in ImmPort. The selected genes continued to be screened for genes associated with prognosis in the TCGA database to get overlapping genes. The results were shown as Venn diagrams.

### Data acquisition and analysis

In TCGA database, transcriptome RNA-Seq data and related clinical data were gathered. Depending on pathological traits, the patient cohort was divided into two groups, LUAD (n=598) and LUSC (n=551). Additionally, we retrieved independent datasets (GSE101929, n=66; GSE19188, n=156; GSE116959, n=68) from the Gene Expression Omnibus (GEO) database (https://www.ncbi.nlm.nih.gov/geo/) to obtain three separate groups of tumor and control samples from NSCLC patients. To investigate the impact of *SPP1* on the pathological stage and patient outcomes, a subgroup analysis was conducted.

### Analysis of receiver operating characteristic (ROC) curve

Using the pROC package in the R programming language, the true and false positive rates data points were obtained, the ROC curve was constructed, and the area of the curve was computed. In the two-dimensional ROC curve, the ordinate and abscissa represented the true positive and true negative rates, respectively.

### Evaluation of logistic regression via univariate and multivariate methods

To assess if *SPP1* could serve as a standalone prognostic indicator in LUAD, univariate and multivariate analyses were used, considering the impact of *SPP1* and clinicopathological characteristics of LUAD. Subsequently, the survival time was assessed using COX regression analysis to determine the impact of various factors and provide the corresponding hazard ratio (HR), while taking into account other potential factors. First, single factor analysis was conducted to assess the extent of the influence of different *SPP1* expression levels on independent variables. Thereafter, we assessed how multiple factors, including *SPP1* expression, affected LUAD prognosis.

### Analysis of individual cells

CancerSEA (http://biocc.hrbmu.edu.cn/CancerSEA/), an online database and tool, facilitates the integrated analysis and interpretation of single-cell transcriptome data to uncover expression patterns of the *SPP1* gene and the underlying biological mechanisms in LUAD.

### Assessment of cancer metastasis

EMTome database (http://ec2-3-231-76-84.compute-1.amazonaws.com/emtome/) was used for the analysis of *SPP1* in lung cancer metastasis. To pinpoint crucial pathways connected to tumor metastasis, Gene Set Enrichment Analysis (GSEA) of gene set relevant to metastasis was carried out. A special GSEA-based enrichment analysis approach called Hallmark pathway enrichment analysis was applied.

### Analysis with the CIBERSORT algorithm

The immune landscape was examined using the CIBERSORT method, and the relationship between *SPP1* expression and immunological performance was assessed. This relationship level was inferred from a large number of tumor transcriptomes of patients with LUAD and immune cell subtypes.

### Timer database analysis

TIMER (https://cistrome.shinyapps.io/timer/), a network server, was used to analyze the relationship between *SPP1* expression and the presence of several immune cells in patients with LUAD, including B lymphocytes, CD8+T lymphocytes, CD4+T lymphocytes, dendritic cells (DCs), macrophages, and neutrophils. We also discovered the connection between *SPP1* overexpression and tumor purity using TIMER’s "Relevant" module and the tumor purity correction section.

### TISIDB database analysis

The TISIDB portal website (http://cis.hku.hk/TISIDB/index.php) was utilized to assess tumor-immune interactions and evaluate the relevance of *SPP1* expression to immunosuppression and immune activation. Moreover, TISIDB was utilized as a valuable resource to explore potential connections between *SPP1* expression and the presence of tumor-infiltrating lymphocytes (TILs) in diverse human cancers. Gene expression profiles were employed to conduct gene set variation analysis, enabling the evaluation of TILs proportions. Spearman test was then used to measure the connection between *SPP1* expression and TILs levels.

### Drug sensitivity analysis

The TCGA-LUAD dataset from the Genomics of Drug Sensitivity in Cancer (GDSC) [50] (https://www.cancerrxgene.org/) database, containing data regarding 510 samples and 367 drugs, was employed for sensitivity analysis. The IC_50_ values of the 367 drugs were compared between the high and low *SPP1* expression groups using the oncoPredict package in R by performing a Wilcoxon rank sum test. Furthermore, Spearman correlation analysis was used to examine the association between the different variables.

### Analysis of data using statistical methods

For comparing two groups, statistical methods, such as t-test and Wilcoxon test, were utilized, while Kruskal-Wallis test was used for comparing several groups. To investigate the survival analysis of the patients, univariate and multivariate logistic regression analyses, as well as Kaplan-Meier (KM) curves that showed the survival analysis curves, were employed. Continuous variables according to whether they had a linear relationship, Spearman’s or Pearson’s correlation coefficients were used to assess the relationship between *SPP1* expression and immune infiltration. All data analyses were performed in R (version 4.0.3) and statistical significance was defined as a p-value of 0.05 or less.

## Supplementary Material

Supplementary Figures

Supplementary Table 1
