# Supplementary material for: Overexpression of SPP1 is a prognostic indicator of immune infiltration in lung adenocarcinoma
Source: Aging (Albany NY). 2024 Feb 7;16(3):2953–77. doi: 10.18632/aging.205526 (PMC10911343; doi:10.18632/aging.205526)
Supplement: Supplementary Figures [file aging-16-205526-s001.pdf]

SUPPLEMENTARY FIGURES

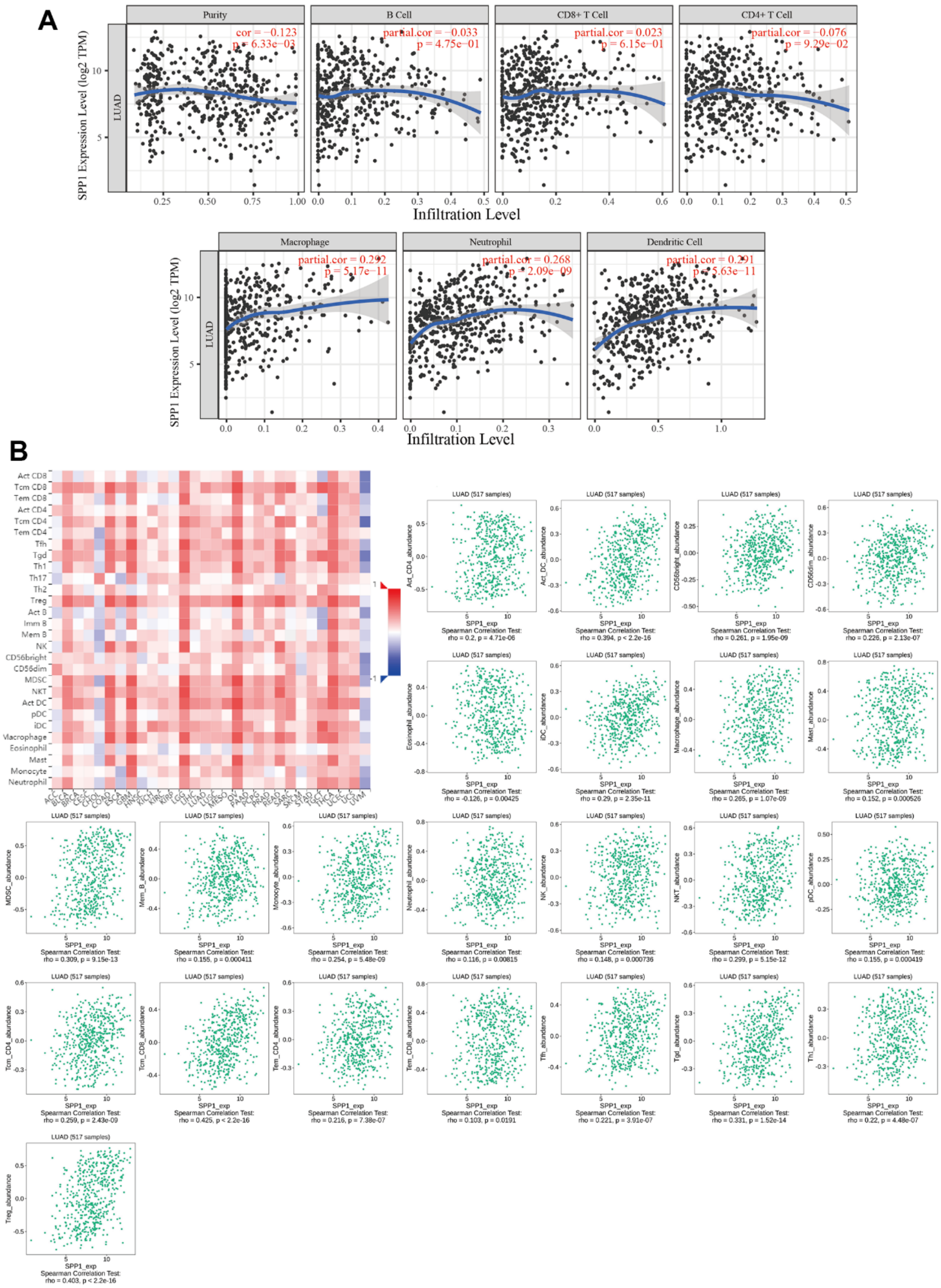

Supplementary Figure 1. Relationship between SPP1 expression and immune cells. (**A**) Negative correlation observed between SPP1 expression and tumor purity, as well as positive correlations between SPP1 expression and dendritic cells, neutrophils, and macrophages in LUAD. (**B**) Relationship between SPP1 and tumor-infiltrating lymphocyte expression in LUAD.

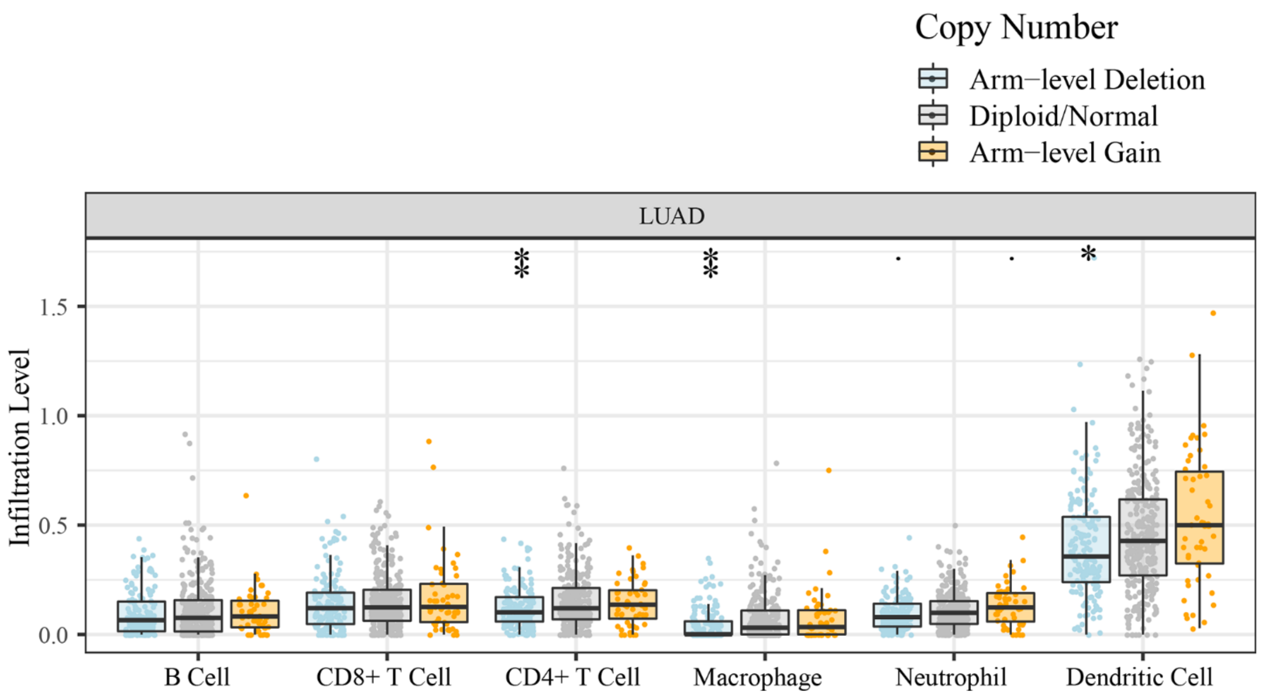

Supplementary Figure 2. The influence of SPP1 copy number variation on the degree of CD4^{+} T cell, macrophage, and dendritic cell infiltration in LUAD. *p<0.05, **p<0.01.
